# Swertiamarin supplementation prevents obesity-related chronic inflammation and insulin resistance in mice fed a high-fat diet

**DOI:** 10.1080/21623945.2021.1906510

**Published:** 2021-04-02

**Authors:** Liang Xu, Dandan Li, Yuqin Zhu, Suili Cai, Xue Liang, Ying Tang, Shengnan Jin, Chunming Ding

**Affiliations:** a School of Laboratory Medicine and Life Sciences, Wenzhou Medical University, Wenzhou Zhejiang, China; b Key Laboratory of Laboratory Medicine, Ministry of Education, Wenzhou Medical University, Wenzhou Zhejiang, China

**Keywords:** Obesity, insulin resistance, oxidative stress, inflammation, swertiamarin

## Abstract

Obesity is characterized by low-grade chronic inflammation, which underlies insulin resistance and non-alcoholic fatty liver disease (NAFLD). Swertiamarin is a secoiridoid glycoside that has been reported to ameliorate diabetes and NAFLD in animal models. However, the effects of swertiamarin on obesity-related inflammation and insulin resistance have not been fully elucidated. Thus, this study investigated the effects of swertiamarin on inflammation and insulin resistance in high-fat diet (HFD)-induced obese mice. C57BL/6 mice were fed a HFD or HFD containing swertiamarin for 8 weeks. Obesity-induced insulin resistance and inflammation were assessed in the epididymal white adipose tissue (eWAT) and livers of the mice. Swertiamarin attenuated HFD-induced weight gain, glucose intolerance, oxidative stress, and insulin resistance, and enhanced insulin signalling in mice. Compared to HFD-fed mice, the swertiamarin-treated mice exhibited increased lipolysis and reduced adipocyte hypertrophy and macrophage infiltration in eWAT. Moreover, swertiamarin alleviated HFD-mediated hepatic steatosis and inflammation by suppressing activation of the p38 MAPK and NF-κB pathways within the eWAT and liver of obese mice. In conclusion, supplementation with swertiamarin attenuated weight gain and hepatic steatosis, and alleviated obesity-associated inflammation and insulin resistance, in obese mice.

## Introduction

Obesity has become one of the most serious global health problems, with an incidence that increases yearly and is associated with a variety of comorbidities, such as type 2 diabetes, non-alcoholic fatty liver disease (NAFLD), and cardiovascular disease [1,2]. Obesity results from an imbalance in energy homoeostasis (calorie consumption vs. energy expenditure), which leads to excessive accumulation of fat in various regions of the body. Chronic low-grade inflammation, which contributes to insulin resistance, has been observed in association with a higher fat mass [3,4]. It is well established that adipocyte-derived adipo-, cyto- and/or chemokines, such as leptin, adiponectin, tumour necrosis factor-alpha (TNF-α), interleukins (ILs), and macrophage chemoattractant protein, are involved in the onset and progression of insulin resistance and NAFLD [5,6]. Moreover, activation of inflammatory pathways in obese subjects, including mitogen-activated protein kinase (MAPK), IκB-kinase β (IκKβ)/nuclear factor-kappa B (NF-κB), and mammalian target of rapamycin (mTOR)/S6 kinase, interferes with insulin signalling [7–9]. A growing body of evidence has revealed that obesity activates inflammation by recruiting immune cells, such as macrophages and T cells, into tissues, which gives rise to the development of insulin resistance [10–12]. Thus, strategies to suppress chronic inflammation in adipose tissue may have the potential to protect against obesity-related insulin resistance, and even attenuate progression to diabetes and steatohepatitis.

Swertiamarin is a secoiridoid glycoside isolated from medicinal plants in the family Gentianaceae [13], which has been reported to protect against several inflammation-associated diseases, including arthritis and diabetes, as well as liver injury [14,15]. These effects are associated with the antioxidant [16,17], antihyperlipidemic [14,18], antinociceptive [19], and free-radical scavenging activities of swertiamarin [20]. Swertiamarin alleviates hyperinsulinemia and hyperglycaemia in diabetic and obese rat models [14,21,22]. In addition, treatment with swertiamarin markedly attenuates NF-κB-related inflammation in animal models of arthritis [23]. Taken together, these results indicate that swertiamarin may contribute to the amelioration of obesity, because obesity is closely related to dyslipidemia and inflammation. Therefore, this study examined the effects of swertiamarin on insulin resistance and chronic inflammation in high-fat diet (HFD)-induced obese mice.

## Materials and Methods

### Animals and diets

Eight-week-old male C57BL/6 mice were purchased from the China National Laboratory Animal Resource Center (Shanghai, China), and randomly divided into four groups fed for 8 weeks as follows: (1) normal chow (NC, n = 6), with 10% of calories from fat (CRF-1; Charles River Laboratories, Wilmington, MA, USA); (2) HFD (n = 6), with 60% of calories from fat (D12492; Research Diets, New Brunswick, NJ, USA); (3) HFD with 0.01% (w/w) swertiamarin (HFD + 0.01% STM, n = 6); or (4) HFD with 0.1% (w/w) swertiamarin (HFD + 0.1% STM, n = 6). All mice were maintained under a 12/12-h light/dark cycle and given free access to food and water. The mice were housed individually in metabolic cages, and daily food and water intake data were collected for 1 week. After an overnight fasting, all mice were killed at 9:00am after deep anaesthesia by peritoneal injection of pentobarbital sodium (50 mg/kg). All animal procedures were performed according to institutional guidelines, and this study was approved by the Wenzhou Medical University Animal Experiment Committee (wydw2019-0941).

### Dosage information

Swertiamarin was purchased from Sigma-Aldrich Corporation (#90,957). We then commissioned Research Diets Inc. to make the high-fat diet (D12492) containing 0.01% and 0.1% swertiamarin for the animal study. It has been demonstrated that swertiamarin at 50 mg/kg protected rats from poloxamer-407-induced hyperlipidaemia [24] and at 100 and 200 mg/kg protected rats against galactosamine-induced hepatic injury [16]. Upon further preliminary experiments, we selected 10 and 100 mg/kg of swertiamarin for the present study [25]. For *in vitro* study, swertiamarin was dissolved in PBS. The cells were treated with swertiamarin at 1, 10, or 50 μg/mL. These concentrations were well tolerated by cultured cells [17,26].

### Cell line culture and treatment

The RAW264.7 (TIB-71; ATCC, Manassas, VA, USA) murine monocytic cell line was cultured in Dulbecco’s modified Eagle’s medium (DMEM; Gibco, Carlsbad, CA, USA) containing 10% foetal bovine serum (FBS; Gibco) until the cells reached 90% confluence. Then, the cells were serum-starved for 6 h and incubated with 10 ng/mL lipopolysaccharide (LPS; Sigma-Aldrich) in the presence of swertiamarin for 16 h. The LPS-induced inflammatory signals were examined by western blotting.

3T3-L1 mouse pre-adipocytes (CL-173, ATCC) were cultured in DMEM supplemented with 10% FBS until confluent. At 2 days post-confluency, the medium was replaced with DMEM containing 0.5 mM methylisobutylxanthine (Sigma-Aldrich), 0.125 mM indomethacin (Sigma-Aldrich), and 1.0 µg/mL insulin (Sigma-Aldrich). After 2 days, the induced cells were cultured in maintenance medium (DMEM containing 10% FBS, 1.0 µg/mL insulin, and 1 nmol/L T3) for 5 days and treated with swertiamarin at the indicated concentrations for 48 h. Oil Red O staining and quantitative real-time polymerase chain reaction (qPCR) were performed to evaluate the lipid content of cells.

### Isolation of peritoneal macrophages and treatments

Mouse peritoneal macrophages were isolated from a male C57BL/6 J mouse, as described previously [27]. The isolated cells were cultured in DMEM containing 10% FBS. The cells were serum-starved for 6 h, co-incubated with 10 ng/mL LPS and swertiamarin for 16 h, and harvested for quantitative real-time PCR analyses.

### ROS production measurements

Intracellular reactive oxygen species (ROS) formation was determined by the 5-(and-6)-chloromethyl-2ʹ, 7ʹ-dichlorodihydrofluorescein diacetateacetylester (CM-H2DCFDA, Invitrogen) fluorescent probe in peritoneal macrophages as described previously [27,28]. Briefly, peritoneal macrophages were pretreated with LPS and swertiamarin for 16 h, and incubated with 10 μM CM-H2DCFDA for 30 min. Fluorescence was measured at an excitation wavelength of 480 nm and an emission wavelength of 525 nm using a Berthold Tristar LB941 (Berthold Technologies, Bad Wildbad, Germany).

### Histological examination and immunohistochemistry

Epididymal white adipose tissue (eWAT) and liver tissues were fixed in 10% formalin. Paraffin wax-embedded sections were stained with haematoxylin and eosin (H&E), or stained immunohistochemically for F4/80, as described previously [29].

### Biochemical analyses and metabolic measurements

The levels of plasma triglycerides (TG), total cholesterol (TC), non-esterified fatty acids (NEFAs), glucose, insulin, aspartate aminotransferase (AST), alanine aminotransferase (ALT), malondialdehyde (MDA), superoxidase dismutase (SOD), and hepatic lipids were measured as described previously [30]. All hepatic lipid levels were normalized relative to liver protein levels.

### Insulin tolerance test and glucose tolerance test

Mice were fasted for 4 h and injected intraperitoneally with 1 U/kg body weight human insulin (Eli Lilly, Kobe, Japan) for the insulin tolerance test (ITT). Blood glucose was measured before and 30, 60, 90, and 120 min after injection. Mice fasted overnight were injected intraperitoneally with 2 g/kg D-glucose solution for the glucose tolerance test (GTT). Blood glucose was again measured before and 30, 60, 90, and 120 min after injection.

### *Insulin signalling* in vivo

Anesthetized mice were injected intraperitoneally with 10 U/kg body weight human insulin for 10 min to detect phosphorylation of Akt (Ser473) *in vivo*. Then, the mice were killed and their tissues were harvested.

### Quantitative real-time PCR analyses

Total RNA was isolated from frozen tissues using TRIzol^TM^ reagent (Invitrogen, Carlsbad, CA, USA), and the concentration was determined with the NanoDrop-5000 spectrophotometer (Thermo Scientific, Waltham, MA, USA). We synthesized cDNA using a high-capacity cDNA reverse transcription kit (Applied Biosystems, Carlsbad, CA, USA) and determined mRNA expression levels via qPCR using SYBR Green, as described previously [31]. The primers used for real-time PCR are listed in Supplementary Table 1.

### Immunoblotting analyses

The eWAT and liver tissues were homogenized in RIPA lysis buffer (Millipore, Billerica, MA, USA) supplemented with protease and phosphatase inhibitors (Sigma-Aldrich). The total protein concentration was determined using a BCA Protein Assay Kit (Pierce, Bonn, Germany). The lysates were blotted overnight at 4°C with primary antibodies (Supplementary Table 2), and then incubated with appropriate secondary antibodies (Cell Signalling Technology, Danvers, MA, USA). The proteins were visualized by chemiluminescence (Millipore) and imaged using a gel imaging system (ChemiDoc™ XRS; Bio-Rad, Hercules, CA, USA). Pixel intensities of the immunoreactive bands were quantified using Quantity One software (ver. 4.5.2; Bio-Rad).

### Statistical analyses

All data are presented as mean ± standard error. A P-value < 0.05 was considered significant. Differences in mean values between two groups were assessed using the two-tailed Student’s *t*-test. Differences in mean values among more than two groups were determined using analysis of variance; if significant, differences between pairs of groups were determined using a Bonferroni post hoc test.

## Results

### Swertiamarin reduces body weight and alleviates insulin resistance in mice fed a HFD

**Figure 1.**
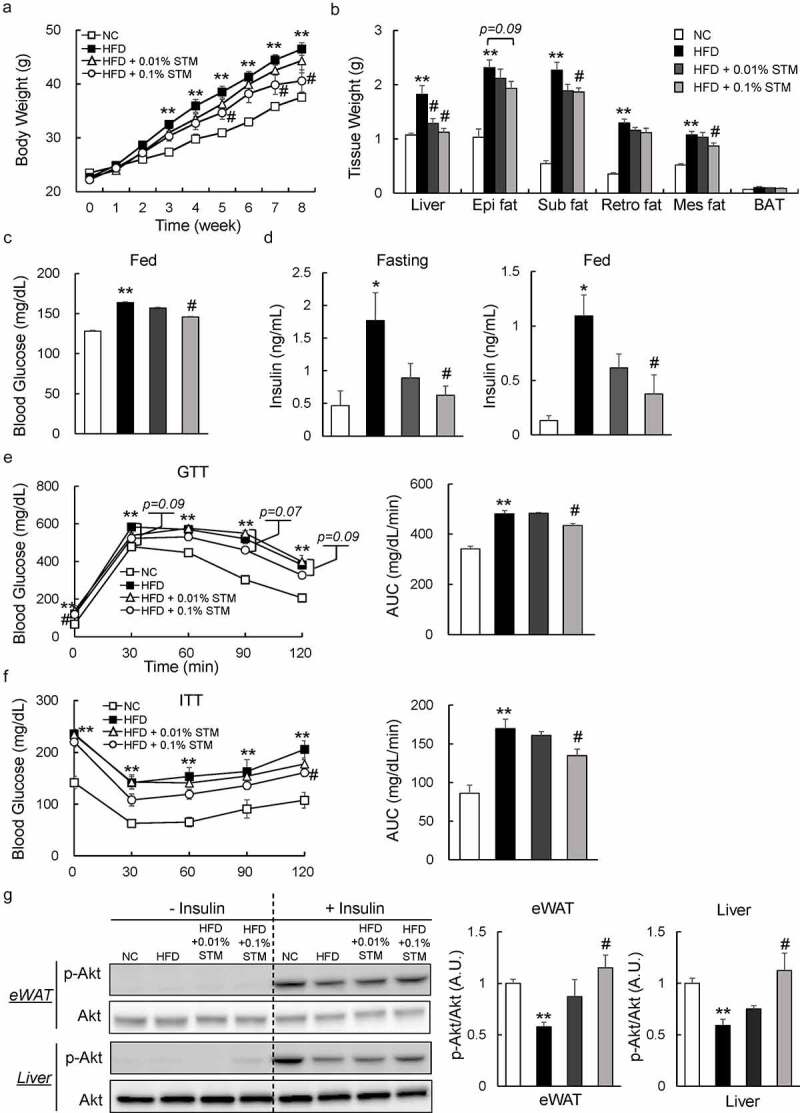
Swertiamarin reduces body weight and insulin resistance in high-fat diet-fed mice. (a) Body weights of the mice. (b) Tissue weights of the mice. (c) Blood glucose levels of the mice under *ad libitum* feeding conditions. (d) Plasma insulin levels of mice fasted for 16 h or under fed conditions. (e) Glucose tolerance test. (f) Insulin tolerance test. (g) Immunoblots of phosphorylated Ser473 Akt (p-Akt) and Akt in the eWAT and liver. Data are mean ± SEM, n = 5–6. *p < 0.05; **p < 0.01 vs. NC; ^#^*p* < 0.05; ^##^*p* < 0.01 vs. HFD. NC, normal chow; HFD, high-fat diet; STM, swertiamarin; Epi, epididymal; Sub, subcutaneous; Retro, retroperitoneal; Mes, mesenteric; BAT, brown adipose tissue; GTT, glucose tolerance test; ITT, insulin tolerance test; eWAT, epididymal white adipose tissue

To investigate the effect of swertiamarin on obesity and insulin resistance, C57BL/6 mice were fed NC, a HFD, or a HFD containing swertiamarin for 8 weeks. Swertiamarin suppressed weight gain (Figure 1(a)) independent of food intake (Table 1). The lower body mass of the 0.1% swertiamarin-treated mice was largely attributable to reduced mass of the liver and WAT deposits, including subcutaneous, epididymal, and mesenteric fat, although the decrease in epididymal fat was not significant (Figure 1(b)). Moreover, plasma TG, TC, and NEFA levels decreased in obese mice in response to swertiamarin, in a dose-dependent manner (Table 1).

**Table 1.** Effects of Swertiamarin on Metabolic Parameters After 8 Weeks of Treatment

	NC	HFD	HFD + 0.01% STM	HFD + 0.1% STM
Food intake (Kcal/kg/day)	410 ± 4.8	439.4 ± 32.3	451.3 ± 21.8	443.8 ± 26.6
Plasma TG (mg/dL)	16.7 ± 2.8	33.3 ± 1.5**	22.7 ± 3.2**^#^**	20.7 ± 3.1**^##^**
Plasma TC (mg/dL)	72.8 ± 2.8	105.4 ± 10.2*	93.2 ± 5.7	68.3 ± 10.4**^#^**
Plasma NEFA (mEq/L)	0.24 ± 0.05	0.43 ± 0.08 *^p=0.08^*	0.26 ± 0.06	0.22 ± 0.05**^#^**
Plasma ALT (IU/L)	7.6 ± 1.7	19.8 ± 3.9*	15.8 ± 2.8	9.3 ± 1.7**^#^**
Plasma AST (IU/L)	3.5 ± 1.0	9.6 ± 3.1 *^p=0.08^*	8.2 ± 2.7	9.4 ± 0.9
Plasma MDA (µmol/L)	1.3 ± 3.4	4.6 ± 7.9 *	3.4 ± 7.0	2.7 ± 3.6 **^#^**
Plasma SOD (U/mL)	30.3 ± 3.9	14.2 ± 2.6 *	19.0 ± 2.4	26.7 ± 4.1 **^#^**

The mice were fasted for 16 h. Data were obtained from 16-week-old mice on different diets. Data are mean ± SEM, n = 5–6. *p < 0.05; **p < 0.01 vs. NC; ^#^*p* < 0.05; ^##^*p* < 0.01 vs. HFD. NC, normal control; HFD, high-fat diet; STM, swertiamarin; TG, triglycerides; TC, total cholesterol (TC); NEFA, nonesterified fatty acid; ALT, alanine aminotransferase; AST, aspartate aminotransferase; MDA, malondialdehyde; SOD, superoxidase dismutase.

We investigated whether swertiamarin can improve insulin resistance in HFD-fed mice. Our data showed that administering swertiamarin normalized blood glucose levels in HFD-fed mice (Figure 1(c)). Furthermore, swertiamarin significantly decreased plasma insulin levels in HFD-fed mice under the fasting and fed conditions (Figure 1(d)). The GTT and ITT data revealed that glucose intolerance and insulin resistance were ameliorated by swertiamarin (Figure 1(e and f)). Consistent with these findings, the insulin-stimulated (10 U/kg) phosphorylation of Akt (Ser473) in eWAT and liver increased in the HFD + STM group relative to the HFD group (Figure 1(g)). Taken together, swertiamarin suppressed weight gain and improved obesity-related insulin resistance by activating insulin signalling.

### Swertiamarin attenuates HFD-induced adipocyte hypertrophy in the eWAT of HFD-fed mice

**Figure 2.**
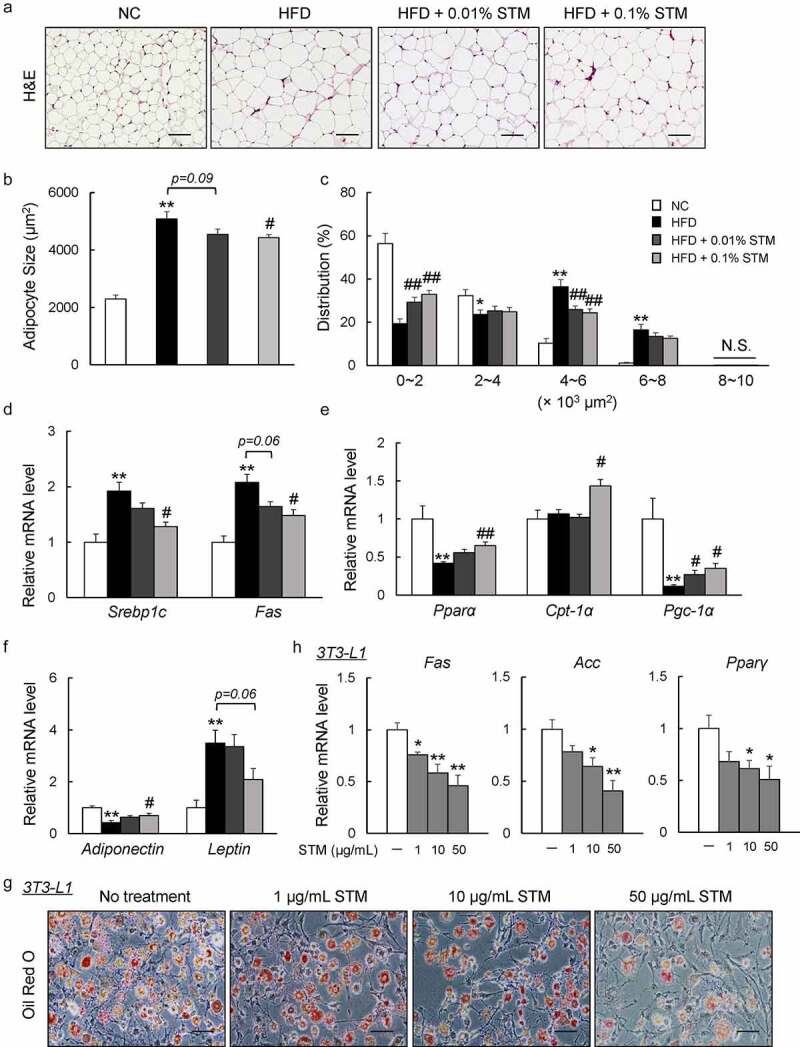
Swertiamarin attenuates HFD-induced adipocyte hypertrophy. (a) Representative H&E-stained eWAT sections. Scale bars = 100 μm. (b) Mean adipocyte size of mice. (c) Distribution of adipocyte size. (d) mRNA expression of lipogenic genes in eWAT. (e) mRNA expression of lipolytic genes in eWAT. (f) mRNA expression of adipokine genes in eWAT. Data are mean ± SEM, n = 5–6. *p < 0.05; **p < 0.01 vs. NC; ^#^*p* < 0.05; ^##^*p* < 0.01 vs. HFD. (g) Oil Red O-stained 3T3-L1 adipocytes. (h) mRNA expression of lipogenic genes in 3T3-L1 adipocytes. Data are mean ± SEM, *n = *5–6. *p < 0.05; **p < 0.01 vs. non-treatment (NT)

Dysfunction in adipocyte morphology, adipokine expression, or metabolism contributes to the development of obesity and insulin resistance [32]. Increased adipocyte size due to fat storage (i.e., adipocyte hypertrophy) was observed in the eWAT of our HFD group, which was attenuated by swertiamarin (Figure 2(a and b)). The difference in mean adipocyte size between the HFD + STM and HFD groups was attributed to the significantly lower proportion of large adipocytes and higher proportion of small adipocytes in the eWAT of HFD + STM mice (Figure 2(c)). These eWAT findings were accompanied by decreased expression of lipogenic genes, such as sterol regulatory element-binding protein-1 c (*Srebp1c*) and fatty acid synthase (*Fas*), and increased expression of lipolytic genes, including peroxisome proliferator-activated receptor α (*Pparα*), carnitine palmitoyltransferase-1α (*Cpt-1α*), and PPAR-gamma coactivator-1α (Figure 2(d and e)). Moreover, the HFD increased the expression of *leptin* and decreased the expression of *adiponectin*. Swertiamarin reversed these changes in adipokine gene expression in the eWAT (figure 2(f)). Lastly, we determined that swertiamarin directly attenuated lipid accumulation and decreased the mRNA levels of lipogenic genes, such as *Fas*, acetyl CoA carboxylase (*Acc*) and *Pparγ*, in 3T3-L1 pre-adipocytes (Figure 1(g and h)). Thus, the decrease in adiposity caused by swertiamarin was largely attributed to decreased lipogenesis and enhanced lipolysis in the adipose tissue of mice fed the HFD.

### Swertiamarin decreases HFD-induced inflammation in WAT

**Figure 3.**
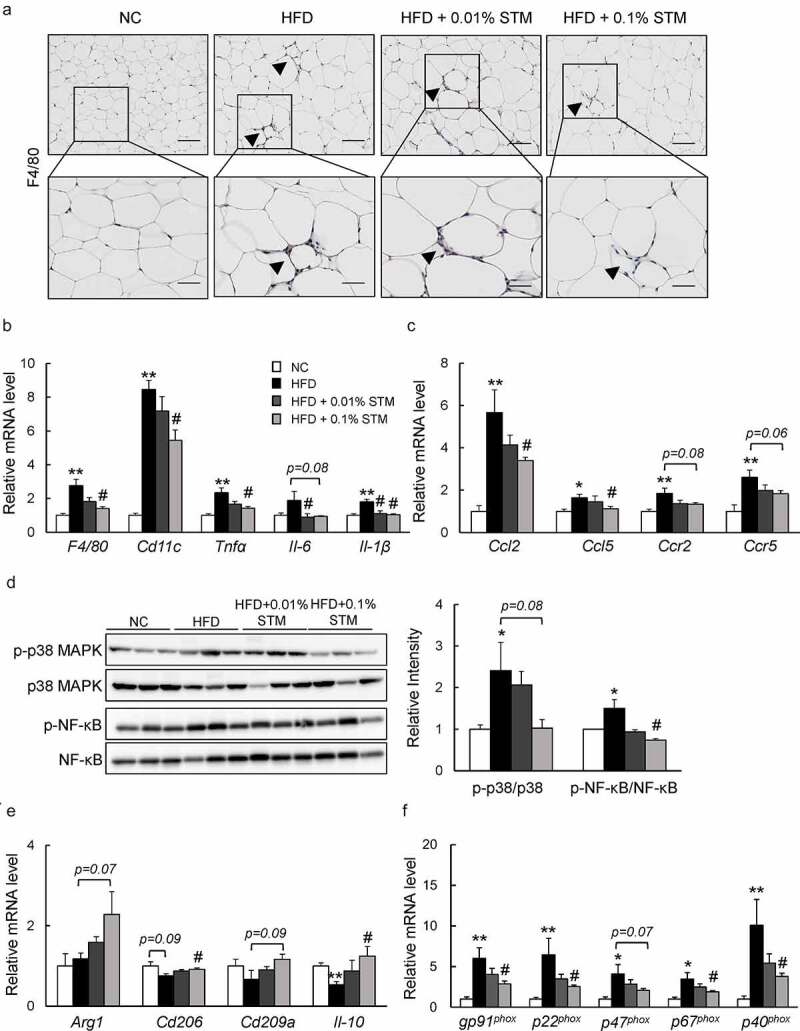
Swertiamarin ameliorates adipose tissue inflammation in HFD-fed mice. (a) Representative F4/80-immunostained eWAT sections. Scale bars = 100 and 50 μm. Arrows indicate crown-like structures. (b) mRNA expression of F4/80 and cytokine genes in eWAT. (c) mRNA expression of chemokine genes in eWAT. (d) Immunoblots of phosphorylated NF-κB p65 (p-NF-κB p65), phosphorylated p38 MAPK (p-p38 MAPK), and their total protein contents in eWAT. (e) mRNA expression of markers for M2 macrophages in eWAT. (f) mRNA expression of NADPH oxidase genes in eWAT. Data are mean ± SEM, n = 5–6. *p < 0.05; **p < 0.01 vs. NC; ^#^*p* < 0.05; ^##^*p* < 0.01 vs. HFD

We also evaluated the effect of swertiamarin on inflammation in adipose tissue. Infiltration of macrophages into hypertrophied adipose tissue, and the occurrence of crown-like structures that were induced by HFD, decreased markedly in response to swertiamarin according to the immunostaining and F4/80 mRNA expression results (Figure 3(a and b)). Consistent with the histological observations, the mRNA levels of inflammatory cytokines, including integrin (*Cd11 c), Tnf-α, Il-6*, and *Il-1β*, decreased in the eWAT of HFD + STM mice compared to HFD mice (Figure 3(b)). Moreover, mRNA expression of proinflammatory chemokines, including chemokine (C-C motif) ligand 2 (*Ccl2), Ccl5*, C-C chemokine receptor 2 (*Ccr2*), and *Ccr5* was also downregulated by swertiamarin (Figure 3(c)). The reduced expression of proinflammatory genes was associated with decreased phosphorylation of MAPK p38 and NF-κB p65 in the eWAT of HFD-fed mice (Figure 3(d)). In contrast, swertiamarin upregulated the mRNA levels of anti-inflammatory genes compared to the HFD group, and also activated macrophage markers (*Arg1, Cd206, Cd209a* and *Il10*) (Figure 3(e)).

In addition, the levels of plasma MDA, an indicator of lipid peroxidation, increased with the HFD, but swertiamarin decreased their levels markedly (Table 1). Moreover, swertiamarin significantly increased the levels of plasma SOD. These findings were observed in association with decreased mRNA expression of the NADPH oxidase subunits in the eWAT of HFD-fed mice (Figure 3(f)). Thus, the anti-inflammatory effects of swertiamarin were at least in part due to the attenuation of oxidative stress in adipose tissue.

### Swertiamarin directly suppresses inflammation in macrophages

**Figure 4.**
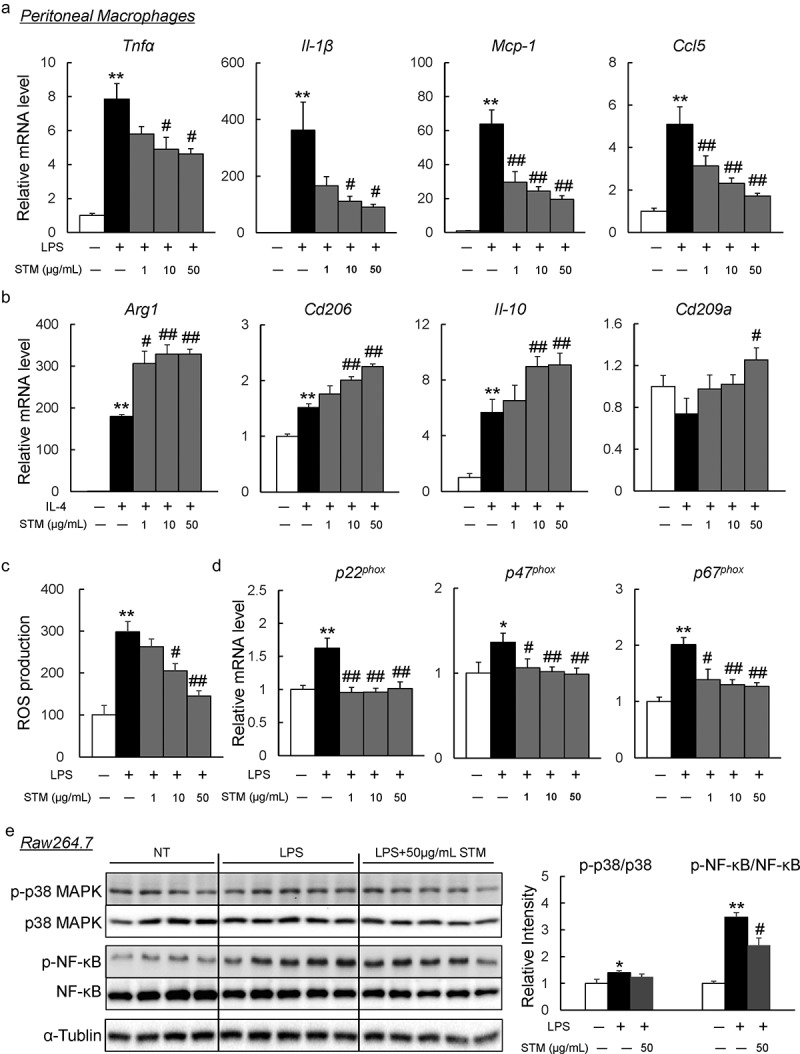
Swertiamarin directly suppresses inflammation in macrophages. (a) mRNA expression of lipopolysaccharide (LPS)-induced M1 markers in peritoneal macrophages. (b) mRNA expression of IL-4-induced M2 markers in peritoneal macrophages. (c) Reactive oxygen species (ROS) production in LPS-treated peritoneal macrophages. (d) mRNA expression of NADPH oxidase subunit genes in LPS-treated peritoneal macrophages. (e) Immunoblot op-p38MAPK, p-ERK, and p-NF-κB in LPS-treated Raw264.7 macrophages. n = 5–6. *p < 0.05; **p < 0.01, vs. non-treatment (NT). ^#^*p* < 0.05; ^##^*p* < 0.01, vs. LPS- or IL4-treated cells

Next, to investigate the effect of swertiamarin on inflammation *in vitro*, we treated the isolated peritoneal macrophages from C57BL/B6 mice with swertiamarin. Swertiamarin decreased the expression of inflammatory cytokines (*Tnf-α* and *Il-1β*) and chemokines (*Ccl2* and *Ccl5*) in LPS-stimulated macrophages (Figure 4(a)). In contrast, swertiamarin enhanced expression of M2 markers (*Arg1, Cd206, IL-10*, and *Cd209a*) in IL-4-stimulated macrophages (Figure 4(b)). LPS-mediated ROS production caused inflammation in macrophages by activating the MAPK and NF-κB pathways. The increase in ROS generation in macrophages was decreased by swertiamarin (Figure 4(c)). These findings were associated with decreased mRNA expression of the NADPH oxidase subunits in LPS-stimulated macrophages (Figure 4(d)). Moreover, swertiamarin significantly suppressed LPS-induced phosphorylation of MAPK p38 and NF-κB p65 in mouse RAW264.7 macrophages (Figure 4(e)). These findings indicate that swertiamarin may alleviate inflammatory signalling through a direct cell autonomous mechanism.

### Swertiamarin suppresses hepatic steatosis in HFD-fed mice

**Figure 5.**
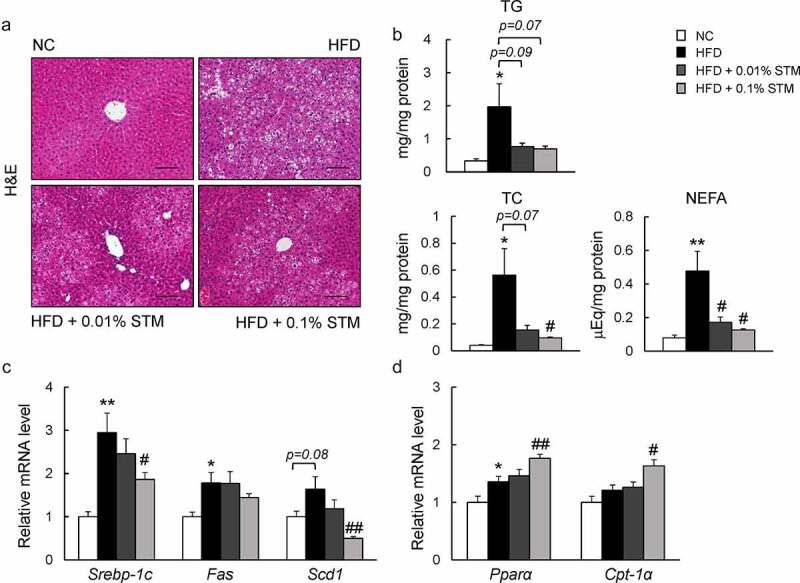
Swertiamarin alleviates HFD-induced hepatic steatosis in mice. (a) Representative H&E-stained liver sections. Scale bars = 100 μm. (b) Liver triglycerides (TG), total cholesterol (TC), and nonesterified fatty acid (NEFA) levels. (c) mRNA expression of lipogenic genes in the liver. (d) mRNA expression of β-oxidation genes in the liver. Data are mean ± SEM, n = 5–6. *p < 0.05; **p < 0.01 vs. NC; ^#^*p* < 0.05; ^##^*p* < 0.01 vs. HFD

A HFD causes hepatic steatosis and inflammation, eventually leading to fatty liver disease [33]. As shown in Figure 5(a) swertiamarin attenuated HFD-induced hepatic steatosis (Figure 5(a)). This finding was observed in association with decreased levels of hepatic TG, TC, and NEFAs (Figure 5(b)). Additionally, the lower levels of plasma AST in the HFD + STM mice compared to the HFD group indicated that swertiamarin alleviated HFD-induced liver damage (Table 1). The decrease in hepatic steatosis was accompanied by decreased expression of lipogenic genes, including *Srebp1c* and stearoyl-CoA desaturase 1 (*Scd1*) (Figure 5(c)), and increased expression of β-oxidation-related genes, such as *Pparα* and *Cpt-1α* (Figure 5(d)).

### Swertiamarin attenuates liver inflammation in HFD-fed mice

**Figure 6.**
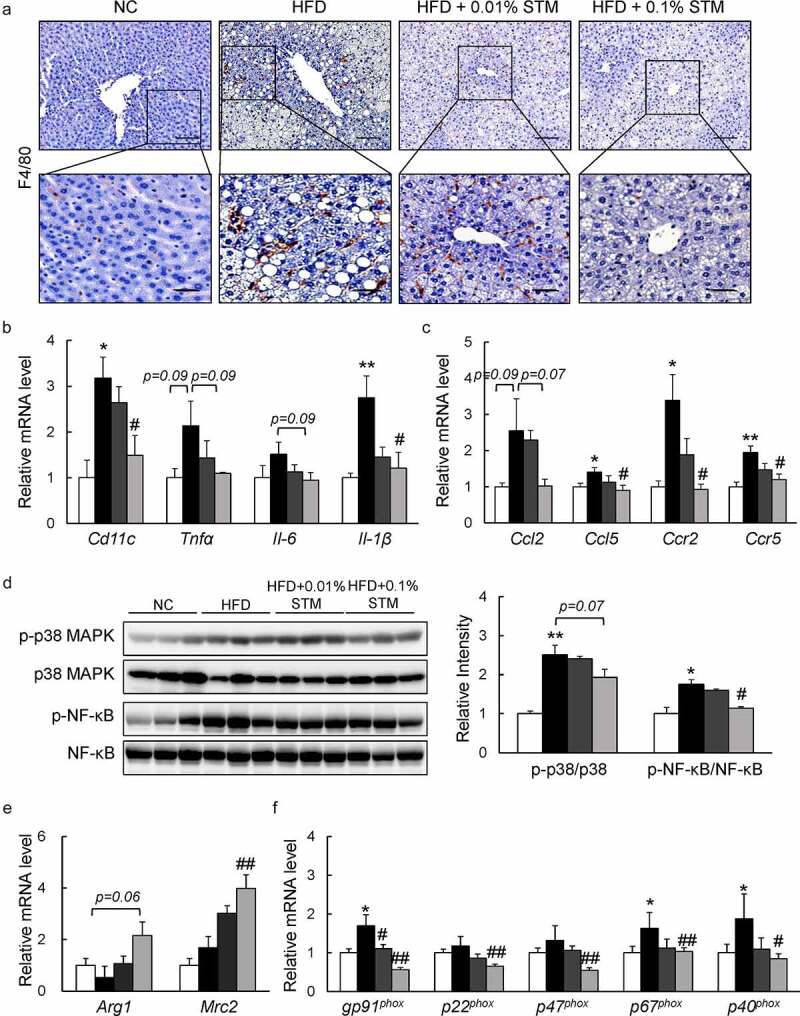
Swertiamarin prevents hepatic inflammation in HFD-fed mice. (a) Representative F4/80-immunostained liver sections. Scale bars = 100 and 50 μm. (b) mRNA expression of cytokine genes in the liver. (c) mRNA expression of chemokine genes in the liver. (d) Immunoblots of p-NF-κB p65, p-p38 MAPK, and their total protein contents in the liver. (e) mRNA expression of M2 macrophage markers in the liver. (f) mRNA expression of NADPH oxidase genes in the liver. Data are mean ± SEM, n = 5–6. *p < 0.05; **p < 0.01 vs. NC; ^#^*p* < 0.05; ^##^*p* < 0.01 vs. HFD

Swertiamarin not only attenuates HFD-induced hepatic steatosis, but also suppresses obesity-related chronic inflammation in the liver. HFD induced macrophage infiltration in the live tissue was markedly reduced by administration of swertiamarin based on F4/80 immunostaining (Figure 6(a)). Meanwhile, the mRNA levels of inflammatory cytokines, including *Cd11 c, Tnf-α, Il-6*, and *Il-1β*, decreased with swertiamarin supplementation in the HFD-fed mice (Figure 6(b)). Swertiamarin also decreased the expression of proinflammatory chemokines and their receptors (e.g., *Ccl2, Ccl5, Ccr2*, and *Ccr5*) in the liver of HFD-fed mice (Figure 6(c)). These findings were associated with attenuation of MAPK p38 and NF-kB p65 expression in the liver of HFD-fed mice (Figure 6(c)). Moreover, swertiamarin enhanced mRNA expression of M2 markers, such as *Arg1* and *Mrc2* (Figure 6(e)). Additionally, swertiamarin suppressed mRNA levels expression of the NADPH oxidase subunits in the liver of HFD-fed mice (figure 6(f)). Thus, swertiamarin protected against HFD-induced chronic inflammation and oxidative stress in the liver of obese mice.

## Discussion

**Figure 7.**
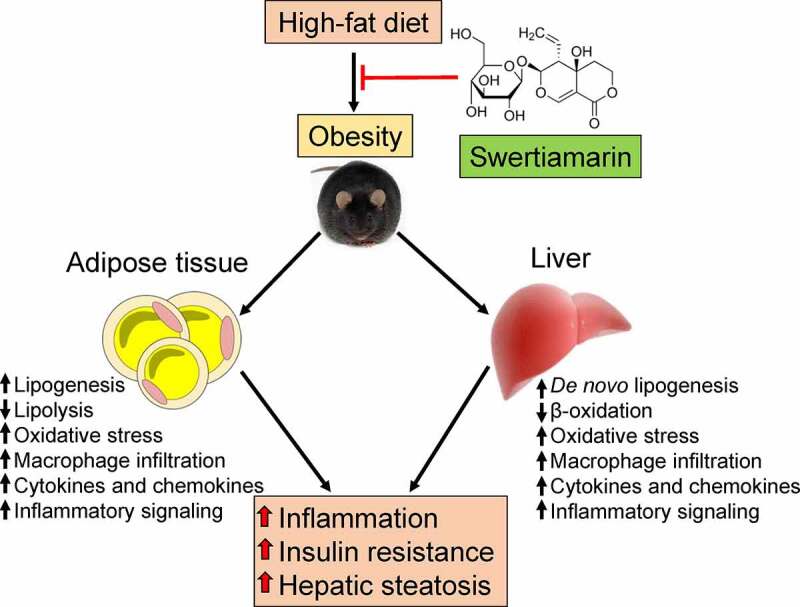
Putative role of swertiamarin in the amelioration of HFD-induced obesity. A high-fat diet induces obesity-related hepatic steatosis and insulin resistance by impairing lipid metabolism and increasing chronic inflammation within adipose tissue and the liver. Administering swertiamarin improves lipid metabolism, decreases cytokine and chemokine expression, and attenuates inflammatory signalling in the fat and liver, thus ameliorating obesity-related chronic inflammation and insulin resistance. HFD, high-fat diet

The results of this study indicate that supplementation with swertiamarin, a secoiridoid glycoside, mitigated HFD-induced weight gain, insulin resistance, chronic inflammation, and hepatic steatosis in mice (Figure 7). Enlargement of the fat mass was suppressed without altering caloric consumption in HFD-fed mice treated with swertiamarin. Moreover, swertiamarin attenuated insulin resistance by enhancing activation of insulin signalling and reducing inflammation in mice fed a HFD. Taken together, these results suggest that swertiamarin has potential for treating obesity and its comorbidities.

Consistent with previous reports [21,22], swertiamarin supplementation improved the systemic glucose tolerance and insulin sensitivity of HFD-fed mice. Moreover, swertiamarin restored insulin-stimulated Akt phosphorylation in the adipose tissue and liver of obese mice. Therefore, these findings suggest that increased adipose tissue and liver insulin signalling contribute to the actions of swertiamarin in regulating glucose homoeostasis.

Along with the reduction of plasma insulin and glucose levels, swertiamarin attenuated lipogenesis and accelerated lipolysis within adipose and liver tissues. In obesity, hyperinsulinemia and hyperglycaemia activate a key enzyme, SREBP-1 c, which regulates *de novo* lipogenesis and contributes to hepatic steatosis [34]. SREBP-1 c is the master regulator of the expression of lipogenic genes, including FAS, ACC, and SCD1 [35,36]. Moreover, treatment with swertiamarin exerts antiadipogenic effects by inhibiting *de novo* fatty acid synthesis, increasing fat breakdown, and promoting fatty acid oxidation in 3T3-L1 adipocytes [37]. Our study revealed that the *Srebp-1 c* mRNA level was downregulated by swertiamarin in the fat and liver tissue of HFD-fed mice, suggesting attenuation of lipogenesis by swertiamarin, partially through decreased *Srebp-1 c* expression. Additionally, swertiamarin has been shown to enhance fatty acid β-oxidation through activation of 3-ketoacyl-coA thiolase, and preventing HFD-induced lipid deposition and hyperlipidaemia [25]. Our findings demonstrated that swertiamarin increased the expression of *Pparα* and *Cpt1α* in the fat and liver, suggesting the promotion of lipolysis and fatty acid oxidation in HFD-fed mice. Together, these results suggest that swertiamarin ameliorates obesity-mediated insulin resistance, and that NAFLD partially depends on decreasing *de novo* lipogenesis and promoting lipolysis in the adipose tissue and liver.

The improved insulin sensitivity in swertiamarin-treated mice was accompanied by decreased oxidative stress and inflammation in the eWAT and liver. Under prolonged overnutrition conditions, excess amounts of ROS can be generated by adipocytes, hepatocytes, and macrophages to overwhelm the cellular antioxidant defence system, resulting in oxidative stress [38]. Accumulation of oxidative stress can activate the NF-κB and MAPK pathways, resulting in increase in the innate immune response and insulin resistance [38]. Here, we found swertiamarin reduced the increase in oxidative stress, as shown by the reduced levels of MDA and NAPDH oxidase expression, and increase in SOD level. Moreover, swertiamarin attenuated ROS generation, suppressed the expression of NADPH oxidase subunits, and suppressed p38 MAPK and NF-κB phosphorylation in cultured macrophages. Thus, the anti-inflammatory effects of swertiamarin are due to the attenuation of the oxidative stress associated with fat accumulation or macrophage infiltration into WAT and/or liver. Our findings are consistent with the anti-oxidative effects of swertiamarin and its preventive effect on insulin resistance and hepatoprotection in obesity [16,39].

Obesity or ectopic fat accumulation induces an innate immune response with subsequent recruitment of immune cells, which ultimately leads to the development of insulin resistance and NAFLD [40,41]. In particular, adipose tissue macrophage (ATM) recruitment and infiltration are crucial in obesity-induced inflammation and insulin resistance [33,42]. In particular, ATM-derived proinflammatory cytokines, such as TNF-α and IL-1β, are similar to classically activated M1-type macrophages that directly contribute to insulin resistance or type 2 diabetes [43]. Conversely, the specific depletion of M1-macrophages restores insulin sensitivity in diet-induced obese mice [44]. These findings suggest that strategies to attenuate macrophage recruitment and infiltration in adipose tissue, restrain M1 macrophage polarization and/or drive M2 activation of macrophages may have the potential to prevent exacerbation of inflammation and insulin resistance, and even progression to NAFLD [45–47].

Our *in vitro* results confirmed that swertiamarin directly suppressed the ROS formation and the proinflammatory activation of macrophages [48]. LPS induces M1-macrophage polarization by stimulating Toll-like receptor 4, which increases NOX-dependent ROS generation and activates MAPK and NF-κB pathways [49]. When swertiamarin was added together with LPS, it suppressed LPS-induced ROS, resulting in attenuation of M1-macrophage stimulation and subsequent NF-κB and MAPK pathways activation. Furthermore, swertiamarin suppressed the infiltration of macrophages into adipose tissue and liver and reduced the expression of M1 mRNA markers, while increased expression of M2 mRNA markers. Therefore, swertiamarin-induced suppression of macrophages infiltration and activation have the potential to prevent the exacerbation of inflammation and insulin resistance.

In conclusion, our data demonstrate that swertiamarin ameliorates obesity and the associated insulin resistance by improving dyslipidemia and attenuating inflammation, which further improves hepatic steatosis. The overall results of our study highlight the potential clinical utility of swertiamarin for preventing obesity and related metabolic disorders, such as insulin resistance, NAFLD, and type 2 diabetes.

## Supplementary Material

Supplemental Material
