# Supplementary material for: Swertiamarin supplementation prevents obesity-related chronic inflammation and insulin resistance in mice fed a high-fat diet
Source: Adipocyte. 2021 Apr 2;10(1):160–73. doi: 10.1080/21623945.2021.1906510 (PMC8023247; doi:10.1080/21623945.2021.1906510)
Supplement: Supplemental Material [file KADI_A_1906510_SM9744.docx]

**Supplementary Table 1-Mice primer sequences.**

| Gene | 5’ Primer | 3’ Primer |
| --- | --- | --- |
| Adiponectin | AGCCGCTTATATGTATCGCTCA | TGCCGTCATAATGATTCTGTTGG |
| Arg1 | CTCCAAGCCAAAGTCCTTAGAG | AGGAGCTGTCATTAGGGACATC |
| Ccl2 | AGGTCCCTGTCATGCTTCTGG CTGCTGCTGGTGATCCTCTTG | CTGCTGCTGGTGATCCTCTTG |
| Ccl5 | TGCCCTCACCATCATCCTCACT | GGCGGTTCCTTCGAGTGACA |
| Ccr2 | ATTCTCCACACCCTGTTTCG | GATTCCTGGAAGGTGGTCAA |
| Ccr5 | CTACCACACCGGGACTGTGAAAC | TCAAACTATGGAAACAGCCCTCATC |
| Cd11c | AAAATCTCCAACCCATGCTG | CACCACCAGGGTCTTCAAGT |
| Cd206 | CAAGGAAGGTTGGCATTTGT | CCTTTCAGTCCTTTGCAAGC |
| Cd209a | CCTGGGAGAGGAAGACTGTG | CTTGCTAGGGCAGGAAGTTG |
| Cpt1α | AAACCCACCAGGCTACAGTG | TCCTTGTAATGTGCGAGCTG |
| F4/80 | CTTTGGCTATGGGCTTCCAGTC | GCAAGGAGGACAGAGTTTATCGTG |
| Fas | AGAGACGTGTCACTCCTGGACTT | GCTGCGGAAACTTCAGAAAAT |
| Gp91phox | TTG GGT CAG CAC TGG CTC TG | TGG CGG TGT GCA GTG CTA TC |
| Il-1β | CTGAACTCAACTGTGAAATGCCA | AAAGGTTTGGAAGCAGCCCT |
| Il-6 | GTTCTCTGGGAAATCGTGGA | GGAAATTGGGGTAGGAAGGA |
| Il-10 | GCTCTTACTGACTGGCATGAG | CGCAGCTCTAGGAGCATGTG |
| Leptin | AAGAAGATCCCAGGGAGGAA | TGATGAGGGTTTTGGTGTCA |
| Mcp1 | AGGTCCCTGTCATGCTTCTGG | CTGCTGCTGGTGATCCTCTTG |
| P22phox | GTCCACCATGGAGCGATGTG | CAATGGCCAAGCAGACGGTC |
| P40phox | GCCGCTATCGCCAGTTCTAC | GCAGGCTCAGGAGGTTCTTC |
| P47phox | GATGTTCCCCATTGAGGCCG | GTTTCAGGTCATCAGGCCGC |
| P67phox | CTGGCTGAGGCCATCAGACT | AGGCCACTGCAGAGTGCTTG |
| Pgc-1α | ATGTGTCGCCTTCTTGCTCT | ATCTACTGCCTGGGGACCTT |
| Pparα | GAGGGTTGAGCTCAGTCA GG | GGTCACCTACGAGTGGCATT |
| Pparγ | GAGGGTTGAGCTCAGTCAGG | GGTCACCTACGAGTGGCATT |
| Scd1 | CATCATTCTCATGGTCCTGCT | CCCAGTCGTACACGTCATTTT |
| Srebp-1c | GGAGCCATGGATTGCACATT | GGCCCGGGAAGTCACTGT |
| Tnfα | AAGCCTGTAGCCCACGTCGTA | GGCACCACTAGTTGGTTGTCTTTG |
| β-actin | AGGCCCAGAGCAAGAGAGGTA | GGGGTGTTGAAGGTCTCAAACA |

**Supplementary Table 2-Antibodies used in immunoblotting.**

| Antibody |  |
| --- | --- |
| anti-phospho-p38 MAPK | Cell signaling (#9211) |
| anti-p38 MAPK | Cell signaling (#9212) |
| anti-phospho-NF-κB p65 | Cell signaling (#3033) |
| anti-NF-κB p65 | Cell signaling (#3034) |
| anti-phospho-IR β | Cell signaling (#3021) |
| anti-IR β | Cell signaling (#3025) |
| anti-phospho-Akt | Cell signaling (#9271) |
| anti-Akt | Cell signaling (#9272) |
| Monoclonal anti-β-actin | Sigma-Aldrich (A5441) |
